# Milk in Gout

**Published:** 1886-02

**Authors:** 


### MILK IN GOUT.

Milk is by far the most useful of any one medicine in the world. It is not always a medicine but, when it is, and used properly it is among the most efficacious. Here is a case that is well told and the same thing is known to many in this country, and it has a lesson worthy to be often repeated. It is not a very profitable one for the Doctor, if persons are only inclined to look to their own health and the food and drink that will aid them in keeping it. A letter from Paris published in London *Truth*, states how the writer was with a friend who had been suffering from an attack of the gout—a disease that as surely comes from fast or improper living as delirium tremens and can be traced either in the individual or his parentage. He was taken on his way to Cannes, and thought himself too ill to recover. There were twinges everywhere. Each essential organ became in turn the seat of the disease, and his spirits were depressed and his brain foggy. An ordinary doctor mildly suggested a lacteal diet, but as the patient hated milk, he refused to accept this regimen. A specialist was then called in. He said, "Meat and wine not excepting Bordeaux—are in your present state rank poison. You must take no tea, coffee or any other stimulant for 10 days. Your food is to be milk every three hours. If you find it too heavy mix it with Hauterive-Vichy. Vary it, too, by having it mixed with onion or leek soups, or with eggs done *a la creme*, I have had patients who were half suffocated with gouty matter in their blood, and others who thought their brains were softening. This treatment cured them in a short time. Milk is food in its most perfect form and should be regarded as a staple ailment by old and young." M. Barthelemy St. Hilaire, who is as old as M. Grevy has lived for years chiefly on milk. He keeps a hornless goat which gives him nearly three quarts a day. An old woman is hired to take the animal out regularly for a walk, and to bring it fresh grass. M. St. Hilaire does not wear a greatcoat however inclement the weather. He walks from Passy to the Senate and Institute, when he has business there, and back; has never ridden in a wheeled vehicle since he was Foreign Minister, is free from every infirmity of old age, and gets up every morning at five o'clock to work at his translation of "Aristotle," of which he has yet seven volumes to get through. He thinks that most of the ills to which the rich are subject come of eating and drinking too much, and trying to have more than their share of enjoyment.

